# Lifestyle and the risk of acute coronary event: a retrospective study of patients after myocardial infarction

**DOI:** 10.3389/fnut.2023.1203841

**Published:** 2023-09-14

**Authors:** Elżbieta Szczepańska, Agnieszka Białek-Dratwa, Katarzyna Filipów, Oskar Kowalski

**Affiliations:** ^1^Department of Human Nutrition, Department of Dietetics, Faculty of Public Health in Bytom, Medical University of Silesia in Katowice, Zabrze, Poland; ^2^Department of Cardiac Surgery, Heart Transplantation and Mechanical Circulatory Support Silesian Centre for Heart Diseases, Zabrze, Poland

**Keywords:** lifestyle, diet, myocardial infarction, eating habits, CVD

## Abstract

**Introduction:**

Unhealthy lifestyle behaviours that may contribute to the development of disorders leading to MI include consuming foods with a high glycaemic load and excessive supply of saturated fats, especially trans fats. Limiting the consumption of simple and refined carbohydrates, such as sweets, sweet drinks, white bread, or white pasta, has a positive effect on the lipid profile by lowering the concentration of triglycerides. Eliminating simple sugars, especially fructose, prevents the deposition of visceral adipose tissue.

**Materials and methods:**

The study included 116 patients of the Silesian Centre for Heart Diseases in Zabrze (SCCS; Poland), with their average age being 59.45 ± 11.54 years, staying in the SCCS due to MI, from March to November 2022. The comprehensive assessment of diet quality included 72 patients: 15 women and 57 men. The research tool was the KomPAN questionnaire for examining dietary views and habits, developed by the Committee on Human Nutrition Science of the Polish Academy of Sciences, evaluating the diet in the year preceding the study. The following three indicators were used to assess the diet quality: pro-Healthy Diet Index (pHDI), non-Healthy Diet Index (nHDI) and Diet Quality Index (DQI).

**Results:**

Most patients ate white wheat bread several times a day (39.66% of patients, with a higher percentage in men than in women – 42.35% vs. 32.26%), and white rice, fine-ground groats and pasta once a week (40.52% of patients, including 41.17% of men and 38.71% of women). Legume seeds were predominantly eaten 1–3 times a month (51.73% of responses, with comparable percentages of men and women, i.e., 51.76% vs. 51.62%), vegetables several times a week (42.25% of responses, including more women than men, i.e., 54.84% vs. 37.64%), and fruit once a day (40.52% of responses, including more men than women: 45.89% vs. 25.81%).

**Conclusion:**

The results of our assessment of individual behaviours of the whole group may indicate errors in the diet. The value of the pro-Healthy Diet Index appears to confirm this fact, while the non-Healthy Diet Index and Diet Quality Index values do not clearly demonstrate its potential adverse impact on health. These limitations of our study may be due to differences in the size of the study population and the size of the population included in the comprehensive diet assessment. Therefore, it seems necessary to conduct further research.

## 1. Introduction

Myocardial infarction (MI) is the ischaemia and necrosis of the heart that occur as a result of reduced or completely prevented blood flow in the coronary vessels (1). It is the main cause of deaths globally (2, 3). Restricted blood flow in the vessels is most often the result of coronary artery disease (CAD) caused by atherosclerotic processes (4). The development of abnormalities contributing to MI can be caused by non-modifiable factors, including male sex, as well as modifiable ones, including an unhealthy lifestyle and its consequences in the form of overweight and excessive adipose tissue content (5–7).

Unhealthy lifestyle behaviours that may contribute to the development of disorders leading to MI include consuming foods with a high glycaemic load and excessive supply of saturated fats, especially trans fats (8, 9). Limiting the consumption of simple and refined carbohydrates, such as sweets, sweet drinks, white bread, or white pasta, has a positive effect on the lipid profile by lowering the concentration of triglycerides (8). Eliminating simple sugars, especially fructose, prevents the deposition of visceral adipose tissue (10). By removing the above-mentioned products from the diet, it is possible to lower blood glucose levels, which reduces cardiovascular risk (8, 11). In turn, the unfavourable effect of saturated fats found in butter, meat products, fatty dairy products, fast foods, and fried foods is conducive to increasing the concentration of cholesterol in blood vessels, which results in the development of atherosclerotic processes leading to CAD (12).

A daily diet for limiting the risk of MI should be rich in complex carbohydrates with high fibre content, unsaturated fats, and plant sterols (12–14). It should include an increased amount of vegetables and fruit, wholegrain cereal products, legumes, low-fat dairy products, unprocessed meat, and fish (15–17). The cardioprotective effect of vegetables and fruit consists in providing fibre and anti-inflammatory compounds to the endothelium of blood vessels (12, 18). Fibre has properties that limit the absorption of cholesterol and lower its concentration in the blood (19). Apart from vegetables, it is also present in wholemeal bread, dark pasta, and groats. Other cholesterol-lowering food ingredients are plant sterols, which are found in vegetable fats, vegetables and sprouts (19). Fish are recommended as the most beneficial source of animal protein that is also believed to have cardioprotective effects (20). Fatty sea fish should be eaten at least twice a week (21). As regards meat consumption, it is better to eat white meat than red meat (22, 23).

In addition to dietary factors, other lifestyle elements that have a real impact on the development of CAD and its ischaemic effects include low physical activity, insufficient sleep, smoking, and alcohol consumption. The duration of daily exercise should be at least 30 min (24). Regular moderate physical activity reduces the risk of MI, MI recurrence, and death caused thereby (25). Not getting enough sleep increases the risk of MI; it is beneficial to sleep at least 7 h per day (26–28). When smoking cigarettes, inhalation of toxic substances induces oxidative stress, damaging the endothelium of blood vessels and resulting in the development of vascular diseases; quitting smoking, in turn, significantly reduces the risk of cardiovascular disease (CVD) (29, 30). While the effect of alcohol consumption on the cardiovascular system remains uncertain, binge drinking has been shown to be significantly associated with the risk of MI (31). On the other hand, some studies indicate that the consumption of small amounts of alcohol may have a potentially beneficial effect on the cardiovascular system (31, 32).

Unhealthy behaviours, including dietary and other habits, increase the risk of CVD. Nevertheless, it is necessary to verify whether lifestyle, apart from its proven effect on the cardiovascular system, may also directly contribute to the occurrence of MI.

The primary aim of this study was to assess the lifestyles of patients 1 year before the onset of MI and to attempt to answer the question whether lifestyle could have been one of its causes. The secondary aim was to investigate whether there were lifestyle differences between women and men and the potential impact of lifestyle on MI.

## 2. Materials and methods

### 2.1. Study population

The study included 116 patients of the Silesian Centre for Heart Diseases in Zabrze (SCCS; Poland), with their average age being 59.45 ± 11.54 years, staying in the SCCS due to MI, from March to November 2022.

The study was carried out in person, in accordance with the principles of the Declaration of Helsinki. The study protocol was approved by the Bioethics Committee of the Medical University of Silesia in Katowice (resolution no. PCN/CBN/0022/KB1/91/21 of July 6, 2021). The patients qualified for participation in the study were informed about the research procedures and gave their informed consent to participate. The criteria for inclusion in the study were: (1) being at least 18 years of age, (2) being hospitalised due to a recent MI (3–30 days after MI; diagnosis according to ICD10: I25.1), (3) functional fitness and motor independence as conditions for independent movement and self-care, and (4) informed consent to participate in the study. The criteria for exclusion from the study were: (1) complicated course of MI, (2) condition preventing independent movement and self-care, (3) psychosocial fitness preventing from independently answering the questions asked, and (4) lack of consent to participate in the study.

The comprehensive assessment of diet quality included 72 patients: 15 women and 57 men, who answered all the questions in the questionnaire. Those patients who could not answer the questions asked, indicating the answer “difficult to assess,” were excluded from the assessment.

### 2.2. Research tool and procedures

The research tool was the KomPAN questionnaire for examining dietary views and habits, developed by the Committee on Human Nutrition Science of the Polish Academy of Sciences, evaluating the diet in the year preceding the study (33). In multicentre studies, the internal reliability (repeatability) of the KomPAN questionnaire was tested, and the results were presented in the publication by Kowalkowska et al. (34). The questionnaire contained four groups of questions, including those concerning eating habits (e.g., the number of meals per day, regularity of eating meals, eating snacks, consumption of salt and sugar), frequency of consumption of certain food products (including potentially healthy and potentially unhealthy ones), lifestyle (alcohol consumption, smoking, sleep time, physical activity), and personal data.

In assessing the frequency of consumption of food products, the following scale was used: (1) never, (2) 1–3 times a month, (3) once a week, (4) a few times a week, (5) once a day, (6) a few times a day.

In the assessment of leisure physical activity, the following criteria were adopted:

- Low: mostly sitting, watching TV, reading the press or books, light housework, walking for 1–2 h a week
- Moderate: walking, cycling, exercising, gardening or other light physical activity for 2–3 h a week
- High: cycling, running, gardening or other sports recreational activities requiring physical effort for more than 3 h a week (33).

The following three indicators were used to assess the diet quality:

- Pro-Healthy Diet Index (pHDI) – taking into account 10 food groups with a potentially beneficial effect on health (wholemeal bread, wholegrain groats and pasta, milk, fermented milk beverages, curd, white meat, fish, legumes, fruit, and vegetables)
- Non-Healthy Diet Index (nHDI) – taking into account 14 groups of food with a potentially adverse impact on health (white bread, white rice, fine-ground groats and pasta, cheese, cured meat and smoked sausages or hot dogs, red meat, fried foods, butter, lard, fast foods, sweets, tinned (jar) meats, sugar-sweetened beverages, energy drinks, alcoholic drinks)
- Diet Quality Index (DQI) – taking into account the 24 food groups mentioned above (35).

The indices were calculated by summing up the frequency of consumption of the 10 food groups (pHDI), 14 food groups (nHDI), and 24 food groups (DQI), while assigning appropriate ranks to the frequencies (never-1, one to three times a month-2, once a week-3, a few times a week-4, once a day-5, a few times a day-6).

The following formulas were used for the calculations:


$$\begin{array}{l} \text{pHDI}\;\left(\text{in}\;\text{points}\right)=\left(100/20\right)\times \text{sum}\;\text{of}\;\text{frequency}\;\text{of}\\ \;\text{consumption}\;\text{of}\;\text{the}\;10\;\text{food}\;\text{groups}\;\left(\text{the}\;\text{pHDI}\;\text{range}\;\text{is}\;0-20\right) \end{array}$$

$$\begin{array}{l} \text{nHDI}\;\left(\text{in}\;\text{points}\right)=\left(100/28\right)\times \text{sum}\;\text{of}\;\text{frequency}\;\\ \text{of}\;\text{consumption}\;\text{of}\;\text{the}\;14\;\text{food}\;\text{groups}\;\left(\text{the}\;\text{nHDI}\;\text{range}\;\text{is}\;0-28\right) \end{array}$$

Interpretation of pHDI, nHDI and DQI scores is presented in Tables 1, 2.

**Table 1:** Interpretation pHDI i nHDI (35).

Intensity of eating characteristics	Range (in points)	
	pHDI	nHDI
Small	0–33	0–33
Medium	34–66	34–66
Big	67–100	67–100

**Table 2:** Interpretation DQI (35).

Range (in points)	Intensity of eating characteristics	Interpretation
−100–−26	High intensity of unhealthy characteristics	The frequency of consumption of food with potentially adverse effects on health is higher than that of food with potentially beneficial effects on health—the effect of the diet is adverse
−25–25	Low intensity of unhealthy characteristics	The frequency of consumption of foods with potentially adverse effects on health is similar to the frequency of consumption of foods with potentially beneficial effects on health—the effect of the diet is neutral
26–100	High intensity of healthy characteristics	The frequency of consumption of foods with potentially beneficial effects on health is higher than that of foods with potentially adverse effects on health—the effect of the diet is beneficial

The DQI was calculated as the sum of all positive-signed pHDI components and all negative-signed nHDI components. Weighting factors were used in the calculations; thus, the share of the 10 pHDI components is the same as the share of the 14 nHDI components. The DQI range is −100 to 100 points.


$$\begin{array}{l} \text{DQI}\;\left(\text{in}\;\text{points}\right)=\left(100/20\right)\times \text{sum}\;\text{of}\;\text{frequency}\;\text{of}\;\\ \text{consumption}\;\text{of}\;\text{the}\;10\;\text{food}\;\text{groups}+\left(-100/28\right)\times \text{sum}\;\\ \text{of}\;\text{frequency}\;\text{of}\;\text{consumption}\;\text{of}\;\text{the}\;14\;\text{food}\;\text{groups} \end{array}$$

The standardised KomPan questionnaire included a question on respondents’ subjective assessment toward their financial situation. The explanation for each level is as follows:

- Financial situation below average - the patient lives modestly, has to budget very frugally on a daily basis
- Average financial situation - the patient has enough for daily living but has to save for more serious purchases
- Financial situation above average - the patient lives well (prosperously), there is enough for a lot without saving.

### 2.3. Statistical analysis

Microsoft Office Word and Microsoft Office Excel programs were used to analyse the collected data. Statistical analysis was performed using Statistica v. 13.3 software (StatSoft Inc., Tulsa, OK, United States). The measured data were represented by mean and standard deviation (X ± SD) as well as minimum and maximum values. Statistical tests were used to analyse the variables for statistical inference. For non-parametric characteristics and two-dimensional tables, Pearson’s chi-squared test was used to compare women and men in terms of lifestyle, including diet, alcohol consumption, leisure activities, and sleep. Cramér’s V coefficient was also calculated. In the result, Cramér’s V coefficient takes values between 0 and + 1 (inclusive); the closer the result is to 0, the weaker the relationship between the examined characteristics, and the closer it is to 1, the stronger the relationship. The statistical significance level of *p* ≤ 0.05 was assumed for all calculations.

## 3. Results

### 3.1. Characteristics of the study population

The characteristics of the study population are presented in Tables 3, 4.

**Table 3:** Socioeconomic characteristics of the studied group of post-MI patients by gender.

		Women *N* = 31		Men *N* = 85		Total *N* = 116	
		*N*	%	*N*	%	*N*	%
Place of residence	City of up to 20.000 inhabitants	3	9.68	5	5.88	8	6.90
	City of 20.000–100.000 inhabitants	8	25.81	18	21.18	26	22.41
	City with over 100.000 inhibitants	20	64.51	55	64.71	75	64.66
	Village	0	0.00	7	8.24	7	6.03
Number of people in the household	1	10	32.26	14	16.47	24	20.69
	2	9	29.03	41	48.24	50	43.10
	3	8	25.81	17	20.00	25	21.55
	4 and more	4	12.91	13	15.29	17	14.65
Number of minors in the household	0	28	90.32	68	80.00	96	82.76
	1	1	3.23	10	11.76	11	9.48
	2	1	3.23	7	8.24	8	6.90
	3	1	3.23	0	0.00	1	0.86
Financial situation	Below the average	2	6.45	2	2.35	4	3.45
	Average	23	74.19	72	84.71	95	81.90
	Above average	6	19.35	9	10.59	15	12.93
	Difficult to assess	0	0.00	2	2.35	2	1.72
Professional work	No. retirement or annuity	19	61.29	31	36.47	50	43.10
	No. unemployed. I run a house	1	3.23	1	1.18	2	1.72
	Yes. Part-time job	0	0.00	2	2.35	2	1.72
	Yes. Permanent employment	10	32.26	47	55.29	57	49.14
	Other	1	3.23	4	4.71	5	4.31
Education	Basic	3	9.68	3	3.53	6	5.17
	Professional	16	51.61	30	35.29	46	39.66
	Medium	6	19.35	33	38.82	39	33.62
	Higher	6	19.35	19	22.35	25	21.55

**Table 4:** Results of anthropometric measurements of the studied group of post-MI patients by gender.

		Women *N* = 31	Men *N* = 85	Total *N* = 116
WHR [cm]	Mean	0.99 ± 0.18	1 ± 0.12	0.99 ± 0.14
	Min-Max	0.74–1.83	0.77–1.83	0.74–1.83
HEIGHT [cm]	Mean	163.65 ± 7.6	174.25 ± 6.46	171.42 ± 8.23
	Min-Max	153–187	156–187	153–187
WEIGHT [kg]	Mean	74.52 ± 18.08	85.39 ± 15.07	82.49 ± 16.57
	Min-Max	54.5–157.7	57.4–123.7	54.5–157.7
BMI [kg/m^2^]	Mean	27.25 ± 3.96	28.03 ± 4.35	27.82 ± 4.24
	Min-Max	22.7–45.1	20.8–39.1	20.8–45.1

The examined group of patients consisted of 116 people, including 31 (26.72%) women and 85 (73.28%) men, most often residing in cities with over 100,000 inhabitants (64.66%). The majority of the patients overall (43.10%) and men (48.24%) were part of two-person households, and women were part of one- (32.26%), two- (29.03%) and three-person (25.81%) households. In addition, the majority of patients’ households did not include any people under the age of 18 (82.76%). Most participants assessed their financial situation as average (81.9%). Almost half of them were professionally active with permanent employment (49.14%), but another large group (43.1%), including more women than men (61.29% vs. 36.47%), were retired or received disability pensions. The largest group of the surveyed patients had basic vocational education (39.66%), including more women (51.61%) than men (35.29%; Table 3).

The average height among participants was 171.42 ± 8.23 cm (163.65 ± 7.6 cm for women and 174.25 ± 6.46 cm for men), and the average body weight was 82.49 ± 16.57 kg (74.52 ± 18.08 kg for women and 85.39 ± 15.07 kg for men). The mean BMI in this population was 27.82 ± 4.24 kg/m2 (27.25 ± 3.96 kg/m2 in women and 28.03 ± 4.35 kg/m2 in men; Table 4).

The patients participating in the study most often did not follow a diet (56.90%), which applies to both women and men (58.06 and 56.47%, respectively). Those who followed a diet most often indicated a diet related to diabetes (16 people, i.e., 32.00%), a low-fat diet (11 people, i.e., 22.00%), or a low-calorie diet (13 people, i.e., 26.00%).

Most patients declared that their eating habits had not changed in recent years and that their eating habits on weekdays compared to weekends differed slightly or did not differ at all (47.42% vs. 44.83%). Women most often answered that their diet on weekdays did not differ from their diet on weekends whatsoever (54.84% of responses), while men that it differed slightly (48.24% of responses).

### 3.2. Lifestyle of the study population

The lifestyle characteristics of the study population are presented in Tables 5–8.

**Table 5:** Selected eating behaviours.

Eating behawior		Women		Men		Total	
		*N* = 31	%	*N* = 85	%	*N* = 116	%
Number of meals *p* = 0.38091 V cr = 0.2136568^*^	1–2	2	6.45	6	7.06	8	6.90
	3	19	61.29	42	49.42	61	52.59
	4–5	10	32.26	37	43.52	47	40.52
Regular meals *p* = 0.14860 V cr = 0.2145346	No	11	35.48	16	18.82	27	23.28
	Yes. Some	10	32.26	46	54.12	56	48.27
	Yes. All	10	32.26	23	27.06	33	28.45
Salting ready meals** p = 0.47691 V cr = 0.1465418	No	19	61.29	45	52.94	64	55.17
	Yes. Sometimes	11	35.48	30	35.29	41	35.34
	Yes. Most foods	1	3.23	10	11.76	11	9.48
Snacking between meals *p* = 0.26787 V cr = 0.2561770	Never	4	12.90	7	8.24	11	9.48
	1–3 times a month	3	9.68	10	11.76	13	11.21
	Once a week	3	9.68	13	15.29	16	13.79
	A few times a week	10	32.26	26	30.59	36	31.03
	Once a day	9	29.03	11	12.94	20	17.24
	A few times a day	1	3.23	13	15.29	14	12.07
	Difficult to assess	1	3.23	5	5.88	6	5.17
Sweetening of hot drinks *p* = 0.68139 V cr = 0.1407063	No	13	41.94	39	45.88	52	44.83
	Yes. 2 or more teaspoons of sugar (or honey)	3	9.68	12	14.12	15	12.93
	Yes. 1 teaspoon of sugar (or honey)	11	35.48	27	31.76	38	32.76
	Yes. Sweeteners	3	9.68	3	3.53	6	5.17
	Difficult to assess	1	3.23	4	4.71	5	4.31

^*^V cr - Cramér’s V coefficient. ^**^Adding salt - Adding salt to prepared foods and sandwiches at the table regardless of where the food was prepared.

**Table 6:** Frequency of consumption of the food groups with potentially beneficial effects on health.

Frequency of consumption		Women		Men		Total	
		*N* = 31	%	*N* = 85	%	*N* = 116	%
Whole wheat bread *p* = 0.32311 V cr = 0.2452231	Never	4	12.90	19	22.35	23	19.83
	1–3 Times a month	1	3.23	9	10.59	10	8.62%
	Once a week	4	12.90	10	11.76	14	12.07
	A few times a week	5	16.13	18	21.18	23	19.83
	Once a day	5	16.13	11	12.94	16	13.79
	A few times a day	5	16.13	11	12.94	16	13.79
	Difficult to assess	7	22.58	7	8.24	14	12.07
Whole grain cereals and pasta *p* = 0.77812 V cr = 0.1671367	Never	3	9.68	12	14.12	15	12.93
	1–3 Times a month	8	25.81	17	20.00	25	21.55
	Once a week	10	32.26	24	28.24	34	29.31
	A few times a month	5	16.13	23	27.06	28	24.14
	Once a day	2	6.45	3	3.53	5	4.31
	A few times a day	0	0.00	1	1.18	1	0.86
	Difficult to assess	3	9.68	5	5.88	8	6.90
Milk *p* = 0.37900 V cr = 0.2350470	Never	6	19.35	13	15.29	19	16.38
	1–3 Times a month	6	19.35	10	11.76	16	13.79
	Once a week	3	9.68	10	11.76	13	11.21
	A few times a week	4	12.90	18	21.18	22	18.97
	Once a day	10	32.26	17	20.00	27	23.28
	A few times a day	1	3.23	13	15.29	14	12.07
	Difficult to assess	1	3.23	4	4.71	5	4.31
Fermented milk beverages *p* = 0.47609 V cr = 0.1975808	Never	4	12.90	5	5.88	9	7.76
	1–3 Times a month	3	9.68	14	16.47	17	14.66
	Once a week	5	16.13	14	16.47	19	16.38
	A few times a week	9	29.04	33	38.83	42	36.2
	Once a day	10	32.26	19	22.35	29	25.00
Curd cheeses *p* = 0.64098 V cr = 0.1917281	Never	2	6.45	6	7.06	8	6.90
	1–3 Times a month	5	16.13	16	18.82	21	18.10
	Once a week	9	29.03	17	20.00	26	22.41
	A few times a week	9	29.03	36	42.35	45	38.79
	Once a day	5	16.13	6	7.06	11	9.48
	A few times a day	1	3.23	4	4.71	5	4.31
White meat dishes *p* = 0.22228 V cr = 0.2452663	Never	0	0.00	1	1.18	1	0.86
	1–3 Times a month	2	6.45	8	9.41	10	8.62
	A few times a week	14	45.16	52	61.18	66	56.90
	Once a week	11	35.48	21	24.71	32	27.59
	A few times a day	1	3.23	0	0.00	1	0.86
	Once a day	3	9.68	3	3.53	6	5.17
Fish *p* = 0.04798 V cr = 0.3104128	Never	2	6.45	2	2.35	4	3.45
	1–3 Times a month	7	22.58	27	31.76	34	29.31
	Once a week	18	58.06	40	47.06	58	47.41
	A few times a week	4	12.90	13	15.29	17	14.66
	Once a day	0	0.00	3	3.53	3	2.59
Legume dishes *p* = 0.01441 V cr = 0.3498260	Never	4	12.90	9	10.59	13	11.21
	1–3 Times a month	16	51.62	44	51.76	60	51.73
	Once a week	6	19.35	27	31.76	33	28.45
	A few times a week	4	12.90	5	5.88	9	7.76
	Once a day	1	3.23	0	0.00	1	0.86
Fruits *p* = 0.04870 V cr = 0.3304078	Never	1	3.23	1	1.18	2	1.72
	1–3 Times a month	1	3.23	6	7.06	7	6.03
	Once a week	0	0.00	7	8.24	7	6.03
	A few times a week	16	51.61	23	27.06	39	33.62
	Once a day	8	25.80	39	45.89	47	40.52
	A few times a day	5	16.13	9	10.59	14	12.07
Vegetables *p* = 0.23313 V cr = 0.2648791	Never	0	0.00	1	1.18	1	0.86
	1–3 Times a month	1	3.23	7	8.24	8	6.90
	Once a week	0	0.00	7	8.24	7	6.03
	A few times a week	17	54.84	33	37.64	50	42.25
	Once a day	8	25.81	23	27.06	31	26.72
	A few times a day	5	16.13	16	18.82	21	18.10

^*^V cr - Cramér’s V coefficient.

**Table 7:** Frequency of consumption of the food groups with potentially adverse effects on health.

Frequency of consumption		Women		Men		Total	
		*N* = 31	%	*N* = 85	%	*N* = 116	%
Wheat bread^**^ p = 0.46370 V cr = 0.2206659	Never	0	0.00	3	3.53	3	2.59
	1–3 Times a month	2	6.45	6	7.06	8	6.90
	Once a week	3	9.68	6	7.06	9	7.76
	A few times a week	9	29.03	11	12.94	20	17.24
	Once a day	6	19.35	18	21.18	24	20.69
	A few times a day	10	32.26	36	42.35	46	39.66
	Difficult to assess	1	3.23	5	5.88	6	5.17
White rice. Small groats and pasta *p* = 0.70602 V cr = 0.1597640	Never	0	0.00	1	1.18	1	0.86
	1–3 Times a month	8	25.81	22	25.88	30	25.86
	Once a week	12	38.71	35	41.17	47	40.52
	A few times a week	9	29.03	26	30.59	35	30.17
	Once a day	2	6.45	1	1.18	3	2.59
Cheese *p* = 0.94272 V cr = 0.1221500	Never	1	3.23	4	4.71	5	4.31
	1–3 Times a month	6	19.35	14	16.47	20	17.24
	Once a week	7	22.58	17	20.00	24	20.69
	A few times a week	11	35.48	34	40.00	45	38.79
	Once a day	3	9.68	11	12.94	14	12.07
	A few times a day	1	3.23	3	3.53	4	3.45
	Difficult to assess	2	6.45	2	2.35	4	3.45
Cold cuts. Sausages *p* = 0.06518 V cr = 0.3197451	Never	0	0.00	1	1.18	1	0.86
	1–3 Times a month	4	12.90	7	8.24	11	9.48
	Once a week	9	29.03	6	7.06	15	12.93
	A few times a week	13	41.94	46	54.12	59	50.86
	Once a day	4	12.90	19	22.35	23	19.83
	A few times a day	1	3.23	6	7.06	7	6.03
Red meat dishes *p* = 0.00631 V cr = 0.3935685	Never	2	6.45	5	5.88	7	6.03
	1–3 Times a month	6	19.35	16	18.82	22	18.97
	Once a week	10	32.26	20	23.53	30	25.86
	A few times a week	5	16.13	37	43.53	42	36.21
	Once a day	1	3.23	5	5.88	6	5.17
	A few times a day	2	6.45	1	1.18	3	2.59
	Difficult to assess	5	16.13	1	1.18	6	5.17
Fried dishes *p* = 0.43434 V cr = 0.2255507	Never	4	12.90	4	4.71	8	6.96
	1–3 Times a month	4	12.90	17	20.00	21	18.26
	Once a week	10	32.26	21	24.71	31	26.96
	A few times a week	10	32.26	32	37.65	42	36.52
	Once a day	0	0.00	5	5.88	5	4.35
	A few times a day	1	3.23	1	1.18	2	1.74
	Difficult to assess	2	6.45	5	5.88	7	6.09
Butter *p* = 0.77678 V cr = 0.1674032	Never	5	16.13	12	14.12	17	14.78
	1–3 Times a month	4	12.90	8	9.41	12	10.43
	Once a week	1	3.23	5	5.88	6	5.22
	A few times a week	5	16.13	20	23.53	25	21.74
	Once a day	8	25.81	14	16.47	22	19.13
	A few times a day	6	19.35	23	27.06	29	25.22
	Difficult to assess	2	6.45	3	3.53	5	4.35
Lard *p* = 0.90507 V cr = 0.1368391	Never	18	58.06	48	56.47	66	57.39
	1–3 Times a month	8	25.81	22	25.88	30	26.09
	Once a week	2	6.45	5	5.88	7	6.09
	A few times a week	0	0.00	2	2.35	2	1.74
	Once a day	1	3.23	1	1.18	2	1.74
	A few times a day	0	0.00	2	2.35	2	1.74
	Difficult to assess	2	6.45	5	5.88	7	6.09
Fast-foods *p* = 0.01871 V cr = 0.3192734	Never	12	38.71	28	32.94	40	34.48
	1–3 Times a month	18	58.06	45	52.94	63	54.31
	Once a week	0	0.00	9	10.59	9	7.76
	A few times a week	1	3.23	3	3.53	4	3.45
Sweets *p* = 0.23762 V cr = 0.2627210	Never	3	9.68%	6	7.06	9	7.76
	1–3 Times a month	12	38.71	24	28.24	36	31.03
	Once a week	5	16.13	11	12.94	16	13.79
	A few times a week	7	22.58	21	24.71	28	24.14
	Once a day	3	9.68	16	18.82	19	16.38
	A few times a day	1	3.23	7	8.24	8	6.90
Tinned (jar) meats *p* = 0.00449 V cr = 0.3828670	Never	8	25.80	13	15.29	21	18.11
	1–3 Times a month	23	74.2	39	45.89	62	53.44
	Once a week	0	0.00	18	21.18	18	15.52
	A few time a week	0	0.00	13	15.29	13	11.21
	Once a day	0	0.00	2	2.35	2	1.72
Sugar-sweetened beverages *p* = 0.29444 V cr = 0.2297476	Never	24	77.42	46	54.12	70	60.34
	A few glasses a month	5	16.13	24	28.24	29	25.00
	A few glasses a week	1	3.23	6	7.06	7	6.03
	1 Glass a day	0	0.00	4	4.71	4	3.45
	2–3 Glasses a day	0	0.00	2	2.35	2	1.72
	Difficult to assess	1	3.23	3	3.53	4	3.45
Energy drinks *p* = 0.76171 V cr = 0.1001624	Never	28	90.32	73	85.88	101	87.07
	A few glasses a month	2	6.45	6	7.06	8	6
	1 Glass a day	0	0.00	3	3.53	3	2.59
	Difficult to assess	1	3.23	3	3.53	4	3.45

^*^V cr - Cramér’s V coefficient. ^**^Whead bread - wheat bread made of refined flour. Toasted bread made of refined wheat flour. Rolls made of refined flour. Baguettes croissants.

**Table 8:** Non-dietary lifestyle elements.

Lifestyle elements		Women		Men		Total	
		*N* = 31	%	*N* = 85	%	*N* = 116	%
Alcohol consumption *p* = 0.23702 V cr = 0.2628	Never	18	58.06	25	29.41	43	37.08
	1–3 Times a month	9	29.03	35	41.18	44	37.93
	Once a week	3	9.68	12	14.12	15	12.93
	A few times a week	1	3.23	8	9.41	9	7.76
	Once a day	0	0	4	4.71	4	3.45
	A few times a day	0	0	1	1.18	1	0.86
Current smoking *p* = 0.76784 V cr = 0.1913	No	27	87.10	72	84.71	99	85.34
	Yes. Less than 5pcs	0	0	2	2.35	2	1.72
	Yes. 5-10pcs	0	0	6	7.06	6	5.17
	Yes. More than10pcs	4	12.90	5	5.88	9	7.76
Smoking in the past *p* = 0.00098 V cr = 0.5952	No	9	29.03	19	22.35	28	24.14
	Yes. Less than 5pcs	2	6.45	3	3.53	5	4.31
	Yes. 5-10pcs	7	22.58	13	15.29	20	17.24
	Yes. More than 10pcs	13	41.94	50	58.82	63	54.31
Hours of sleep *p* = 0.93352 V cr = 0.0610	6 or less hours	7	22.58	22	25.88	29	25.00
	More than 6. less than 9 h	20	64.52	53	62.35	73	62.93
	9 or more hours	3	9.6	6	7.06	9	7.76
	Hard to define	1	3.23	4	4.71	5	4.31
Hours in front of the TV or computer *p* = 0.59192 V cr = 0.1998	Less than 2 h	4	12.9	19	22.36	23	19.89
	2–4 h	26	83.87	60	70.60	86	74.14
	More than 4 h	1	3.23	6	7.06	7	6.03
Physical activity in leisure time *p* = 0.59824 V cr = 0.1272	Low	12	38.71	31	36.47	43	37.07
	Moderate	18	58.06	44	51.76	62	53.45
	High	1	3.23	10	11.76	11	9.48

*V cr - Cramér’s V coefficient.

Patients tended to eat 3 meals a day—this number was indicated by 52.59% of them, including more women (61.29%) than men (49.42%). The tendency to eat certain meals at fixed times was reported by 48.27% of patients, including more men (54.12%) than women (32.26%). Furthermore, in women, not eating meals at fixed times (35.48%) and eating all meals at fixed times (32.26%) were indicated with a similar frequency.

Snacking between meals several times a week was reported by 31.03% of patients, with comparable percentages between women and men (32.26% vs. 30.59%). Most patients declared that they do not add salt to ready meals, as this answer was given by 55.17% of them, including more women (61.29%) than men (52.94%), and that they do not sweeten hot drinks, with 44.83% of the responses, including 41.94% in women and 45.88% in men (Table 5).

The frequency of consumption of wholegrain bread was most often several times a week or never (19.83% each), while for wholegrain cereal products (groats, pasta), it was several times a week (29.31% of responses). The above-mentioned groups of products were more frequently consumed by women than men, with the frequency still being unsatisfactory.

The most popular frequency of milk consumption was once a day, indicated by 23.28% of patients, including more women (32.26%) than men (20%). 21.18% of men drank milk several times a week. Fermented milk beverages and curd were in most cases consumed several times a week (34.48 and 37.93% of responses, respectively), with this frequency of consumption being indicated by more men than women (Table 6).

The most popular frequency of consumption of white meat was several times a week: this answer was given by 56.9% of patients, including more men than women (61.18% vs. 45.16%). Fish was most often eaten once a week, which was the case for 50.00% of patients, including more women and men (58.06% vs. 47.06%).

Legume seeds were predominantly eaten 1–3 times a month (51.73% of responses, with comparable percentages of men and women, i.e., 51.76% vs. 51.62%), vegetables several times a week (42.25% of responses, including more women than men, i.e., 54.84% vs. 37.64%), and fruit once a day (40.52% of responses, including more men than women: 45.89% vs. 25.81%). Most women (51.61%) ate fruit several times a week. The frequency of consumption is unsatisfactory for both of these groups of products (Table 6).

Most patients ate white wheat bread several times a day (39.66% of patients, with a higher percentage in men than in women—42.35% vs. 32.26%), and white rice, fine-ground groats and pasta once a week (40.52% of patients, including 41.17% of men and 38.71% of women).

The most frequently indicated frequency of consumption of products that are a source of animal fats, including cheese, cured meat, sausages and hot dogs, as well as red meat, was several times a week (38.79, 50.86 and 36.21% of responses, respectively), these answers were given by more men than women for each of the above-mentioned product groups (Table 7).

Most patients ate fried foods several times a week (36.52%, with more responses among men than women, i.e., 37.65% vs. 32.26%), and used butter as an addition to bread or in food preparation several times a day (25.22% of responses, including 27.06% of men and 19.35% of women). 25.81% of women used butter once a day. Lard was typically not used as an addition to bread or in food preparation, as indicated by 57.39% of patients, including comparable percentages in women and in men. Fast foods as well as canned and pickled products were consumed by most patients with a frequency of 1–3 times a month (54.31 and 53.44% of responses, respectively), with the former being eaten with similar frequency by women and men, and the latter more often by women than men.

31.03% of patients indicated that they usually ate sweets 1–3 times a month, with a higher percentage in women than in men (38.71% vs. 28.24%). Most patients declared that they did not consume sugar-sweetened cold drinks or energy drinks (60.34 and 87.07%, respectively). In both product groups, the percentage of women indicating these answers was higher than the percentage of men (77.42% vs. 54.12 and 90.32% vs. 85.88%; Table 7).

The largest group of patients indicated that they consumed alcohol 1–3 times a month (37.93%, including more men than women) or did not consume alcohol at all (37.08%, including more women than men). 85.34% of patients (87.10% of women and 84.71% of men) did not smoke cigarettes, but in the past the percentage of non-smokers was only 24.14%, (29.03% of women and 22.35% of men). Among those who had smoked tobacco in the past, the most frequent amount was more than 10 cigarettes a day (50.86%, with a higher percentage in men than in women—54.12% vs. 41.94%). Most patients slept 7–8 h a day, with similar percentages in women (64.42%) and men (62.35%), and spent 2–4 h a day watching TV or using a computer, with a higher percentage in women than in men (83.87% vs. 70.60%). 53.45% of patients, including 58.06% of women and 51.75% of men, assessed their physical activity as moderate, and as many as 37.07%, including comparable percentages of women and men (38.71% vs. 36.47%), as low (Table 8).

### 3.3. Diet quality assessment

**Figure 1:**
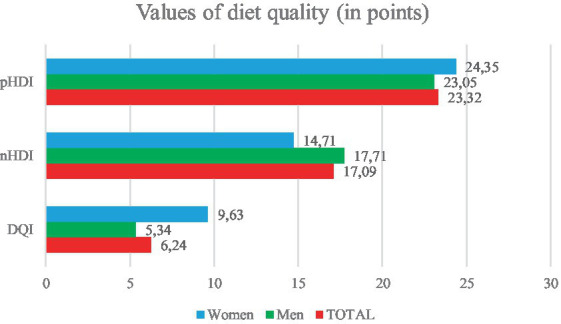
Comprehensive diet quality assessment.

The results of a comprehensive assessment of the diet quality of the patients participating in the study are presented in Figure 1.

Both in the case of the pro-Healthy Diet Index (pHDI) and the non-Healthy Diet Index (nHDI), the index values indicated a low intensity of beneficial and adverse characteristics of nutrition in women and men. With regard to the general Diet Quality Index (DQI), due to the similar intensity of both pro-health and unhealthy characteristics, it can be assumed that the diet used by women and men most likely had a neutral impact on health (Figure 1).

## 4. Discussion

The results of many studies allow to link the reduction of CVD risk with a healthy diet, adequate physical activity, being a non-smoker or having quitted smoking, and maintaining a normal body weight (5, 8, 9). A healthy lifestyle can prevent many cases of coronary artery disease, ischaemic strokes, but also premature deaths associated with heart disease (12, 13, 17, 30).

The analysis of the results of this study showed that patients most often consumed 3 meals a day, and they ate only some of those meals regularly. 1/3 of patients ate snacks between meals, approx. 45% added salt to ready meals, and approx. 55% sweetened hot drinks. Similar results were obtained by Mikulska et al., who assessed the eating habits of people with and without CVD. According to the authors, in both groups, the most common errors involved eating irregularly, i.e., having an improper number of meals during the day and snacking between them (36). In turn, Pachocka et al., in their study of the impact of lifestyle on the level of nutrition in elderly people with metabolic syndrome, showed that 36% of people sweetened their beverages and 65.6% added salt to their food (37). As research shows, the quality of diet, including the number and regularity of meals, snacking, or the use of salt and sugar, is strongly associated with an increased risk of morbidity and even mortality due to CVD. Thus, current guidelines recommend eating 4–5 meals regularly, limiting the consumption of beverages and foods with added sugars, and choosing and preparing foods with little or no salt (13, 17, 24).

Studies show that a plant-based diet is associated with better cardiovascular health. Diets that are rich in vegetables, fruits, legumes, whole grains, and nuts contain protective ingredients, including dietary fibre and antioxidants, which reduce the risk of CVD. They are also low in calories and devoid of saturated fats and added sugars that increase this risk (5, 13, 19, 34, 36). Our research has shown an insufficient frequency of consumption of wholegrain cereals, legumes, fruits and vegetables, with differences in the consumption of these products by women and men only regarding legumes and fruits. Similar unhealthy behaviours were also found in the studies by Mikulska et al. (36) and Mrazova et al. (38).

As shown in our research, products potentially beneficial to health, such as milk, fermented milk beverages or curd, were consumed with a varied but insufficient frequency; however, no significant differences in the frequency of their consumption by women and men were found. Milk and milk products are a source of complete protein, calcium, magnesium, potassium, B vitamins, and vitamin D (24). Fermented milk beverages deserve special attention in this group, due to the fact that they contain healthy microflora. As a result of the research, it was found that fermented products—owing to the content of calcium and magnesium, vitamins D and K, as well as bioactive peptides and bioactive lipids, including CLA phospholipids—reduce the risk of CVD and metabolic diseases, lower blood pressure, have an anti-inflammatory effect, reduce the risk of developing diabetes, and positively affect cholesterol levels (5). Although milk and dairy products contain milk fat, which is a source of saturated fatty acids, associated with an increased risk of CVD, meta-analyses of both prospective cohort studies and randomised controlled trials have shown that its effect is inconclusive. The results of these studies indicate that the consumption of dairy products in general, both full-fat and low-fat, does not increase the risk of CVD (39).

Our research has shown a satisfactory frequency of consumption of white meat. This correct behaviour is in line with the scientific evidence supporting the health benefits of its consumption. As demonstrated by Lupoli et al., the results of their study show, for the first time, a strong and inverse relationship between white meat consumption and all-cause mortality, and a neutral relationship with cardiovascular morbidity and mortality. This highlights the importance of differentiating meat types due to their impact on health, and suggests that white meat may be a healthier alternative to red and processed meat (22). Another study, which evaluated the relationship between the consumption of white meat and the occurrence of cardiometabolic risk factors, showed that only the consumption of lean white meat appears to have a potentially beneficial effect in terms of these risk factors (23).

The frequency of fish consumption among the patients surveyed in our study was insufficient, as 14.66% of them ate fish several times a week, and 47.41% once a week. Differences in the frequency of consumption of fish by women and men were found. Low consumption of fish was also indicated in studies conducted by Mikulska et al. (36), Mrazowa et al. (38), Krupa-Kotara et al. (40). Meanwhile, as shown by Petermann-Rocha et al., eating fish instead of meat is associated with a lower risk of adverse cardiovascular effects (20). The protective effect of fish consumption was further demonstrated by the results of a study by Khatun et al. (41). In addition, a study by Mohan et al. showed an association between a minimum fish consumption of 175 g (approx. 2 servings) per week and a lower risk of major CVD and mortality in post-CVD patients. However, such a relationship has not been demonstrated in general populations (21).

Taking into account the frequency of consumption of unhealthy products by the patients participating in our study, with the exception of fast foods and lard, it can be concluded that they are included in the diet more often than they should. Similar conclusions were drawn by Krupa-Kotara et al. (40). At the same time, differences in the frequency of consumption by women and men were observed only in the case of red meat and fast foods. Products such as cheese, sausages, red meat, butter, and fast foods are a significant source of saturated fatty acids. Meanwhile, the results of numerous studies indicate that limiting the supply of saturated fats in the diet has a positive effect in terms of reducing the cardiovascular risk (5, 9, 13). Furthermore, research suggests that restricting the consumption of saturated fats for at least 2 years results in a potentially significant reduction in total cardiovascular events. In addition, replacing energy from saturated fats with polyunsaturated fats or complex carbohydrates appears to be a correct healthcare strategy (9). The above-mentioned products are also a natural source of sodium, which in combination with adding salt to dishes or meals at the table significantly increases the risk of hypertension and its consequences. Therefore, it seems necessary to implement education to help understand the impact of saturated fats, trans fats, omega-3 and omega-6 polyunsaturated fats, and monounsaturated fats on the risk of atherosclerotic cardiovascular disease (ASCVD) and complications related thereto (12).

Besides nutrition, other lifestyle elements-such as alcohol consumption, smoking, lack of physical activity, or insufficient rest-are recognised as modifiable CVD risk factors (5, 29, 42). The results of our research has shown that most patients consumed alcohol 1–3 times a month or did not consume alcohol at all. 85.34% of patients (87.10% of women and 84.71% of men) did not smoke cigarettes in the last year, while in the past the percentage of non-smokers was only 24.14%, (29.03% of women and 22.35% of men). The largest group who smoked tobacco in the past were people who smoked more than 10 cigarettes/day. Guidelines from many scientific societies, including the American Heart Association (AHA), the American College of Cardiology (ACC) and the National Lipid Association (NLA) or the European Society of Cardiology (ESC), recommend limiting alcohol consumption to a maximum of 100 g per week. Alcohol consumption above this limit reduces life expectancy (17, 29, 43). According to the guidelines, quitting smoking is also recommended, as smoking is strongly and independently associated with ASCVD. Quitting smoking is potentially the most effective preventive method entailing a significant decrease in (recurring) myocardial infarction and mortality (5, 43). As shown in a study by Wang et al., patients with CVD who are smokers are at an increased risk of all-cause mortality, CVD and cancer, and this risk decreases significantly after they quit smoking. These data provide further strong evidence to support the recommendation to quit smoking to prevent premature death among patients with CVD (30).

The analysis of our research results has shown that patients most often slept 7–8 h a day, with comparable percentages in women (64.42%) and men (62.35%). According to Daghlas et al., who studied the relationship between sleep duration and MI, people who slept less than 6 h a day had a 20% higher risk of MI. Thus, their prospective observational analyses confirmed that short sleep is a potential risk factor for MI (26). Similarly, in a study assessing the impact of sleep habits on the risk of acute myocardial infarction (AMI) and coronary artery disease (CAD) in a group of post-AMI patients and in a control group, Lian et al. showed that insufficient sleep is an important risk factor for both AMI risk and CAD severity. In addition, late sleeping is associated with an increased risk of AMI (27).

In our study, 53.45% of patients, including 58.06% of women and 51.75% of men, assessed their physical activity as moderate, and as many as 37.07%, including comparable percentages of women and men (38.71% vs. 36.47%), as low. Furthermore, patients usually spent 2–4 h a day watching TV or using a computer, with a higher percentage in women than in men (83.87% vs. 70.60%). In addition, in research by Mrozowa et al. (38), who assessed CVD risk factors in patients hospitalised after MI, and by Pachocka et al., who studied the impact of lifestyle on the level of nutrition in patients with metabolic syndrome, it was shown that physical activity undertaken was insufficient, and a significant proportion of patients did not undertake any activity at all. The guidelines of scientific societies recommend to reduce the time spent in a sedentary position and to undertake at least light activity during the day in order to reduce all-cause mortality and CVD morbidity and mortality (43). Physical activity reduces the risk of numerous adverse clinical events, regardless of age and gender. There is an inverse relationship between moderate to severe physical activity and all-cause mortality, cardiovascular morbidity and mortality, and the incidence of type 2 diabetes. The risk reduction persists throughout the range in terms of amounts of activity. At the same time, the guidelines indicate that physical activity requires an individual approach in terms of frequency, intensity, duration, and type (43–45).

In our study, the intensity of pro-health diet characteristics was assessed as low (pHDI = 23.32), with no significant differences observed between the intensity of these characteristics in women and in men (pHDI = 24.45 and pHDI = 33.05, respectively). This may indicate that the patients did not pay special attention to the appropriate composition of their meals. Their diets were potentially poor in sources of dietary fibre, polyunsaturated fatty acids, antioxidant vitamins, etc., which could have contributed to the lack of cardioprotective effect resulting from the consumption of certain food groups. As in the case of the pro-Healthy Diet Index, the intensity of diet characteristics with potentially adverse effects on health was assessed as low (nHDI = 17.09); however, the group of women was characterised by a lower intensity of unhealthy characteristics (nHDI = 14.71) compared to men (nHDI = 17.71). The Diet Quality Index indicates a low intensity of both unhealthy and healthy characteristics (DQI = 6.24), in both men (DQI = 5.34) and women (DQI = 9.63); thus, it most likely had a neutral impact on their health.

The results of our own and other authors’ studies clearly show that in view of dietary errors made by people with and without CVD, there is a constant need to implement preventive measures, including education on nutrition, aimed at increasing patients’ awareness of the need to modify their lifestyles.

## 5. Conclusion

1. The assessment of the diet of patients before the onset of MI indicates that they in fact made certain dietary errors, mainly in the insufficient frequency of consumption of wholegrain products, milk and fermented beverages, fish, vegetables and fruit, as well as the excessive frequency of consumption of products with a high content of saturated fat, such as cheese, red meat, cured meat and sausages, or butter.
2. The results of our assessment of individual behaviours of the whole group may indicate errors in the diet. The value of the pro-Healthy Diet Index appears to confirm this fact, while the non-Healthy Diet Index and Diet Quality Index values do not clearly demonstrate its potential adverse impact on health. These limitations of our study may be due to differences in the size of the study population and the size of the population included in the comprehensive diet assessment. Therefore, it seems necessary to conduct further research.
3. The assessment of non-dietary lifestyle elements indicates a positive change in the reduction of the percentage of smokers. Unhealthy behaviours, on the other hand, concern the number of hours spent sleeping, time spent in front of the TV or computer, or too little physical activity.
4. Few differences in terms of lifestyle were found between women and men.
5. Lifestyle may have been one of the causes of MI.

## 6. Strengths and limitations of the study

Our study is one of the few in which the diets of patients with a history of MI in the year preceding the study was comprehensively assessed, using the KomPAN standardised questionnaire for examining dietary views, eating habits, and lifestyle. This allowed not only for a detailed analysis of individual behaviours of patients, but also for a comprehensive assessment of the quality of their diet.

The results of our assessment of individual behaviours of the whole group may indicate errors in the diet. The value of the pro-Healthy Diet Index appears to confirm this fact, while the non-Healthy Diet Index and Diet Quality Index values do not clearly demonstrate its potential adverse impact on health. These limitations of our study may be due to differences in the size of the study population and the size of the population included in the comprehensive diet assessment. Therefore, it seems necessary to conduct further research.

## Data availability statement

The raw data supporting the conclusions of this article will be made available by the authors, without undue reservation.

## Ethics statement

The study was carried out in person, in accordance with the principles of the Declaration of Helsinki. The study protocol was approved by the Bioethics Committee of the Medical University of Silesia in Katowice (resolution no. PCN/CBN/0022/KB1/91/21 of July 6, 2021). Written informed consent was obtained from the participant/patient(s).

## Author contributions

ES: conceptualisation, methodology, analysis of results, writing - preparing and writing an original draft - reviewing and editing. KF, AB-D, and ES: research. AB-D: statistical design, review. OK: supervision. All authors contributed to the article and approved the submitted version.

## Conflict of interest

The authors declare that the research was conducted in the absence of any commercial or financial relationships that could be construed as a potential conflict of interest.

## Publisher’s note

All claims expressed in this article are solely those of the authors and do not necessarily represent those of their affiliated organizations, or those of the publisher, the editors and the reviewers. Any product that may be evaluated in this article, or claim that may be made by its manufacturer, is not guaranteed or endorsed by the publisher.
