# Supplementary material for: Lifestyle and the risk of acute coronary event: a retrospective study of patients after myocardial infarction
Source: Front Nutr. 2023 Sep 14;10:1203841. doi: 10.3389/fnut.2023.1203841 (PMC10538544; doi:10.3389/fnut.2023.1203841)
Supplement: Supplementary file 1 [file Data_Sheet_1.PDF]

frontiers

Supplementary Material

Lifestyle and the Risk of Acute Coronary Event: A Retrospective Study of Patients after Myocardial Infarction

Elżbieta Szczepańska[1], Agnieszka Białek-Dratwa[1]*, Katarzyna Filipów [2] and Oskar Kowalski [1,2,]

[1] Department of Human Nutrition, Department of Dietetics, Faculty of Health Sciences in Bytom, Medical University of Silesia in Katowice, ul. Jordana 19, 41-808 Zabrze, Poland; abialek@sum.edu.pl
[2] Department of Cardiology, Congenital Heart Diseases and Electrotherapy, Silesian Center for Heart Diseases, Zabrze, Poland, k.filipow@sccs.pl (KF)|okowalski@sum.edu.pl (OK)

*** Correspondence:**
abialek@sum.edu.pl (A.B.-D.); Tel.: +48-(0-32)-275-51-95

1 Supplementary Figures and Tables

Table 1 Interpretation pHDI i nHDI [35]

Intensity of eating characteristics	Range (in points)	
	pHDI	nHDI
small	0-33	0-33
medium	34-66	34-66
big	67-100	67-100

Table 2 Interpretation DQI [35]

**Range (in points)**	**Intensity of eating characteristics**	**Interpretation**
-100 – -26	High intensity of unhealthy characteristics	The frequency of consumption of food with potentially adverse effects on health is higher than that of food with potentially beneficial effects on health—the effect of the diet is adverse
-25 – 25	Low intensity of unhealthy characteristics	The frequency of consumption of foods with potentially adverse effects on health is similar to the frequency of consumption of foods with potentially beneficial effects on health—the effect of the diet is neutral
26 – 100	High intensity of healthy characteristics	The frequency of consumption of foods with potentially beneficial effects on health is higher than that of foods with potentially adverse effects on health—the effect of the diet is beneficial

Table 3 Socioeconomic characteristics of the studied group of post-MI patients, by gender

		Women N=31		Men N=85		Total N=116	
		N	%	N	%	N	%
**Place of residence**	city of up to 20,000 inhabitants	3	9,68	5	5,88	8	6,90
	city of 20,000-100,000 inhabitants	8	25,81	18	21,18	26	22,41
	city with over 100,000 inhibitants	20	64,51	55	64,71	75	64,66
	village	0	0,00	7	8,24	7	6,03
**Number of people in the household**	1	10	32,26	14	16,47	24	20,69
	2	9	29,03	41	48,24	50	43,10
	3	8	25,81	17	20,00	25	21,55
	4 and more	4	12,91	13	15,29	17	14,65
**Number of minors in the household**	0	28	90,32	68	80,00	96	82,76
	1	1	3,23	10	11,76	11	9,48
	2	1	3,23	7	8,24	8	6,90
	3	1	3,23	0	0,00	1	0,86
**Financial situation**	below the average	2	6,45	2	2,35	4	3,45
	average	23	74,19	72	84,71	95	81,90
	above average	6	19,35	9	10,59	15	12,93
	difficult to assess	0	0,00	2	2,35	2	1,72
**Professional work**	no, retirement or annuity	19	61,29	31	36,47	50	43,10
	no, unemployed, I run a house	1	3,23	1	1,18	2	1,72
	yes, part-time job	0	0,00	2	2,35	2	1,72
	yes, permanent employment	10	32,26	47	55,29	57	49,14
	other	1	3,23	4	4,71	5	4,31
**Education**	basic	3	9,68	3	3,53	6	5,17
	professional	16	51,61	30	35,29	46	39,66
	medium	6	19,35	33	38,82	39	33,62
	higher	6	19,35	19	22,35	25	21,55

Table 4 Results of anthropometric measurements of the studied group of post-MI patients, by gender

		**Women N=31**	**Men N=85**	**Total N=116**
**WHR [cm]**	Mean	0,99 ± 0,18	1 ± 0,12	0,99 ± 0,14
	min-max	0,74 - 1,83	0,77 - 1,83	0,74 - 1,83
**HEIGHT [cm]**	Mean	163,65 ± 7,6	174,25 ± 6,46	171,42 ± 8,23
	min-max	153 - 187	156 - 187	153 - 187
**WEIGHT [kg]**	Mean	74,52 ± 18,08	85,39 ± 15,07	82,49 ± 16,57
	min-max	54,5 - 157,7	57,4 - 123,7	54,5 - 157,7
**BMI [kg/m^2]**	Mean	27,25 ± 3,96	28,03 ± 4,35	27,82 ± 4,24
	min-max	22,7 - 45,1	20,8 - 39,1	20,8 - 45,1

Table 5 Selected eating behaviours

Eating behawior		Women		Men		Total	
		N=31	%	N=85	%	N=116	%
**Number of meals** p=0,38091 V cr=0,2136568*	1-2	2	6,45	6	7,06	8	6,90
	3	19	61,29	42	49,42	61	52,59
	4-5	10	32,26	37	43,52	47	40,52
**Regular meals** p=0,14860 V cr=0,2145346	no	11	35,48	16	18,82	27	23,28
	yes, some	10	32,26	46	54,12	56	48,27
	yes, all	10	32,26	23	27,06	33	28,45
**Salting ready meals**** p=0,47691 V cr=0,1465418	no	19	61,29	45	52,94	64	55,17
	yes, sometimes	11	35,48	30	35,29	41	35,34
	yes, most foods	1	3,23	10	11,76	11	9,48
**Snacking between meals** p=0,26787 V cr=0,2561770	never	4	12,90	7	8,24	11	9,48
	1-3 times a month	3	9,68	10	11,76	13	11,21
	once a week	3	9,68	13	15,29	16	13,79
	a few times a week	10	32,26	26	30,59	36	31,03
	once a day	9	29,03	11	12,94	20	17,24
	a few times a day	1	3,23	13	15,29	14	12,07
	difficult to assess	1	3,23	5	5,88	6	5,17
**Sweetening of hot drinks** p=0,68139 V cr=0,1407063	no	13	41,94	39	45,88	52	44,83
	yes, 2 or more teaspoons of sugar (or honey)	3	9,68	12	14,12	15	12,93
	yes, 1 teaspoon of sugar (or honey)	11	35,48	27	31,76	38	32,76
	yes, sweeteners	3	9,68	3	3,53	6	5,17
	difficult to assess	1	3,23	4	4,71	5	4,31

*V cr - Cramér’s V coefficient

**Adding salt - Adding salt to prepared foods and sandwiches at the table regardless of where the food was prepared.

Table 6 Frequency of consumption of the food groups with potentially beneficial effects on health

**Frequency of consumption**		**Women**		**Men**		**Total**	
		**N=31**	**%**	**N=85**	**%**	**N=116**	**%**
**Whole wheat bread** p=0,32311 V cr=0,2452231	never	4	12,90	19	22,35	23	19,83
	1-3 times a month	1	3,23	9	10,59	10	8,62
	once a week	4	12,90	10	11,76	14	12,07
	a few times a week	5	16,13	18	21,18	23	19,83
	once a day	5	16,13	11	12,94	16	13,79
	a few times a day	5	16,13	11	12,94	16	13,79
	difficult to assess	7	22,58	7	8,24	14	12,07
**Whole grain cereals and pasta** p=0,77812 V cr=0,1671367	never	3	9,68	12	14,12	15	12,93
	1-3 times a month	8	25,81	17	20,00	25	21,55
	once a week	10	32,26	24	28,24	34	29,31
	a few times a month	5	16,13	23	27,06	28	24,14
	once a day	2	6,45	3	3,53	5	4,31
	a few times a day	0	0,00	1	1,18	1	0,86
	difficult to assess	3	9,68	5	5,88	8	6,90
**Milk** p=0,37900 V cr=0,2350470	never	6	19,35	13	15,29	19	16,38
	1-3 times a month	6	19,35	10	11,76	16	13,79
	once a week	3	9,68	10	11,76	13	11,21
	a few times a week	4	12,90	18	21,18	22	18,97
	once a day	10	32,26	17	20,00	27	23,28
	a few times a day	1	3,23	13	15,29	14	12,07
	difficult to assess	1	3,23	4	4,71	5	4,31
**Fermented milk beverages** p=0,47609 V cr=0,1975808	never	4	12,90	5	5,88	9	7,76
	1-3 times a month	3	9,68	14	16,47	17	14,66
	once a week	5	16,13	14	16,47	19	16,38
	a few times a week	9	29,04	33	38,83	42	36,2
	once a day	10	32,26	19	22,35	29	25,00
**Curd cheeses** p=0,64098 V cr=0,1917281	never	2	6,45	6	7,06	8	6,90
	1-3 times a month	5	16,13	16	18,82	21	18,10
	once a week	9	29,03	17	20,00	26	22,41
	a few times a week	9	29,03	36	42,35	45	38,79
	once a day	5	16,13	6	7,06	11	9,48
	a few times a day	1	3,23	4	4,71	5	4,31
**White meat dishes** p=0,22228 V cr=0,2452663	never	0	0,00	1	1,18	1	0,86
	1-3 times a month	2	6,45	8	9,41	10	8,62
	a few times a week	14	45,16	52	61,18	66	56,90
	once a week	11	35,48	21	24,71	32	27,59
	a few times a day	1	3,23	0	0,00	1	0,86
	Once a day	3	9,68	3	3,53	6	5,17
**Fish** p=0,04798 V cr=0,3104128	never	2	6,45	2	2,35	4	3,45
	1-3 times a month	7	22,58	27	31,76	34	29,31
	once a week	18	58,06	40	47,06	58	47,41

	a few times a week	4	12,90	13	15,29	17	14,66
	once a day	0	0,00	3	3,53	3	2,59
**Legume dishes** p=0,01441 V cr=0,3498260	never	4	12,90	9	10,59	13	11,21
	1-3 times a month	16	51,62	44	51,76	60	51,73
	once a week	6	19,35	27	31,76	33	28,45
	a few times a week	4	12,90	5	5,88	9	7,76
	once a day	1	3.23	0	0.00	1	0.86
**Fruits** p=0,04870 V cr=0,3304078	never	1	3,23	1	1,18	2	1,72
	1-3 times a month	1	3,23	6	7,06	7	6,03
	once a wek	0	0,00	7	8,24	7	6,03
	a few times a week	16	51,61	23	27,06	39	33,62
	once a day	8	25,80	39	45,89	47	40,52
	a few times a day	5	16,13	9	10,59	14	12,07
**Vegetables** p=0,23313 V cr=0,2648791	never	0	0,00	1	1,18	1	0,86
	1-3 times a month	1	3,23	7	8,24	8	6,90
	once a week	0	0,00	7	8,24	7	6,03
	a few times a week	17	54,84	33	37,64	50	42,25
	once a day	8	25,81	23	27,06	31	26,72
	a few times a day	5	16,13	16	18,82	21	18,10

*V cr - Cramér’s V coefficient

Table 7 Frequency of consumption of the food groups with potentially adverse effects on health

**Frequency of consumption**		**Women**		**Men**		**Total**	
		**N=31**	**%**	**N=85**	**%**	**N=116**	**%**
**Wheat bread****<br>p=0,46370<br>V cr=0,2206659	never	0	0,00	3	3,53	3	2,59
	1-3 times a month	2	6,45	6	7,06	8	6,90
	once a week	3	9,68	6	7,06	9	7,76
	a few times a week	9	29,03	11	12,94	20	17,24
	once a day	6	19,35	18	21,18	24	20,69
	a few times a day	10	32,26	36	42,35	46	39,66
	difficult to assess	1	3,23	5	5,88	6	5,17
**White rice, small groats and pasta**<br>p=0,70602<br>V cr=0,1597640	never	0	0,00	1	1,18	1	0,86
	1-3 times a month	8	25,81	22	25,88	30	25,86
	once a week	12	38,71	35	41,17	47	40,52
	a few times a week	9	29,03	26	30,59	35	30,17
	once a day	2	6,45	1	1,18	3	2,59
**Cheese**<br>p=0,94272<br>V cr=0,1221500	never	1	3,23	4	4,71	5	4,31
	1-3 times a month	6	19,35	14	16,47	20	17,24
	once a week	7	22,58	17	20,00	24	20,69
	a few times a week	11	35,48	34	40,00	45	38,79
	once a day	3	9,68	11	12,94	14	12,07
	a few times a day	1	3,23	3	3,53	4	3,45
	difficult to assess	2	6,45	2	2,35	4	3,45
**Cold cuts, sausages**<br>p=0,06518<br>V cr=0,3197451	never	0	0,00	1	1,18	1	0,86
	1-3 times a month	4	12,90	7	8,24	11	9,48
	once a week	9	29,03	6	7,06	15	12,93
	a few times a week	13	41,94	46	54,12	59	50,86
	once a day	4	12,90	19	22,35	23	19,83
	a few times a day	1	3,23	6	7,06	7	6,03
**Red meat dishes**<br>p=0,00631<br>V cr=0,3935685	never	2	6,45	5	5,88	7	6,03
	1-3 times a month	6	19,35	16	18,82	22	18,97
	once a week	10	32,26	20	23,53	30	25,86
	a few times a week	5	16,13	37	43,53	42	36,21
	once a day	1	3,23	5	5,88	6	5,17
	a few times a day	2	6,45	1	1,18	3	2,59
	difficult to assess	5	16,13	1	1,18	6	5,17
**Fried dishes**<br>p=0,43434<br>V cr=0,2255507	never	4	12,90	4	4,71	8	6,96
	1-3 times a month	4	12,90	17	20,00	21	18,26
	once a week	10	32,26	21	24,71	31	26,96
	a few times a week	10	32,26	32	37,65	42	36,52
	once a day	0	0,00	5	5,88	5	4,35
	a few times a day	1	3,23	1	1,18	2	1,74
	difficult to assess	2	6,45	5	5,88	7	6,09
**Butter**<br>p=0,77678<br>V cr=0,1674032	never	5	16,13	12	14,12	17	14,78
	1-3 times a month	4	12,90	8	9,41	12	10,43
	once a week	1	3,23	5	5,88	6	5,22

	a few times a week	5	16,13	20	23,53	25	21,74
	once a day	8	25,81	14	16,47	22	19,13
	a few times a day	6	19,35	23	27,06	29	25,22
	difficult to assess	2	6,45	3	3,53	5	4,35
**Lard**<br>p=0,90507<br>V cr=0,1368391	never	18	58,06	48	56,47	66	57,39
	1-3 times a month	8	25,81	22	25,88	30	26,09
	once a week	2	6,45	5	5,88	7	6,09
	a few times a week	0	0,00	2	2,35	2	1,74
	once a day	1	3,23	1	1,18	2	1,74
	a few times a day	0	0,00	2	2,35	2	1,74
	difficult to assess	2	6,45	5	5,88	7	6,09
**Fast-foods**<br>p=0,01871<br>V cr=0,3192734	never	12	38,71	28	32,94	40	34,48
	1-3 tmes a month	18	58,06	45	52,94	63	54,31
	once a week	0	0,00	9	10,59	9	7,76
	a few times a week	1	3,23	3	3,53	4	3,45
**Sweets**<br>p=0,23762<br>V cr=0,2627210	never	3	9,68%	6	7,06	9	7,76
	1-3 times a month	12	38,71	24	28,24	36	31,03
	once a week	5	16,13	11	12,94	16	13,79
	a few times a week	7	22,58	21	24,71	28	24,14
	ance a day	3	9,68	16	18,82	19	16,38
	a few times a day	1	3,23	7	8,24	8	6,90
**Tinned (jar) meats**<br>p=0,00449<br>V cr=0,3828670	never	8	25,80	13	15,29	21	18,11
	1-3 times a month	23	74,2	39	45,89	62	53,44
	once a week	0	0,00	18	21,18	18	15,52
	a few time a week	0	0,00	13	15,29	13	11,21
	once a day	0	0,00	2	2,35	2	1,72
**Sugar-sweetened beverages**<br>p=0,29444<br>V cr=0,2297476	never	24	77,42	46	54,12	70	60,34
	a few glasses a month	5	16,13	24	28,24	29	25,00
	a few glasses a week	1	3,23	6	7,06	7	6,03
	1 glass a day	0	0,00	4	4,71	4	3,45
	2-3 glasses a day	**0**	0,00	2	2,35	2	1,72
	difficult to assess	1	3,23	3	3,53	4	3,45
**Energy drinks**<br>p=0,76171<br>V cr=0,1001624	never	28	90,32	73	85,88	101	87,07
	a few glasses a month	2	6,45	6	7,06	8	6
	1 glass a day	0	0,00	3	3,53	3	2,59
	difficult to assess	1	3,23	3	3,53	4	3,45

*V cr - Cramér’s V coefficient

** whead bread - wheat bread made of refined flour, toasted bread made of refined wheat flour, rolls made of refined flour, baguettes, croissants

Table 8 Non-dietary lifestyle elements

Lifestyle elements		Women		Men		Total	
		N=31	%	N=85	%	N=116	%
**Alcohol consumption** p=0,23702 V cr=0,2628	never	18	58,06	25	29,41	43	37,08
	1-3 times a month	9	29,03	35	41,18	44	37,93
	once a week	3	9,68	12	14,12	15	12,93
	a few times a week	1	3,23	8	9,41	9	7,76
	once a day	0	0	4	4,71	4	3,45
	a few times a day	0	0	1	1.18	1	0.86
**Current smoking** p=0,76784 V cr=0,1913	no	27	87,10	72	84,71	99	85,34
	yes, less than 5pcs	0	0	2	2,35	2	1,72
	yes, 5-10pcs	0	0	6	7,06	6	5,17
	yes, more than10pcs	4	12,90	5	5,88	9	7,76
**Smoking in the past** p=0,00098 V cr=0,5952	no	9	29,03	19	22,35	28	24,14
	yes, less than 5pcs	2	6,45	3	3,53	5	4,31
	yes, 5-10pcs	7	22,58	13	15,29	20	17,24
	yes, more than 10pcs	13	41,94	50	58,82	63	54,31
**Hours of sleep** p=0,93352 V cr=0,0610	6 or less hours	7	22,58	22	25,88	29	25,00
	more than 6, less than 9 hours	20	64,52	53	62,35	73	62,93
	9 or more hours	3	9,6	6	7,06	9	7,76
	hard to define	1	3,23	4	4,71	5	4,31
**Hours in front of the TV or computer** p=0,59192 V cr=0,1998	less than 2 hours	4	12,9	19	22,36	23	19,89
	2-4 hours	26	83,87	60	70,60	86	74,14
	more than 4 hours	1	3,23	6	7,06	7	6,03
**Physical activity in leisure time** p=0,59824 V cr=0,1272	low	12	38,71	31	36,47	43	37,07
	moderate	18	58,06	44	51,76	62	53,45
	high	1	3,23	10	11,76	11	9,48

*V cr - Cramér’s V coefficient

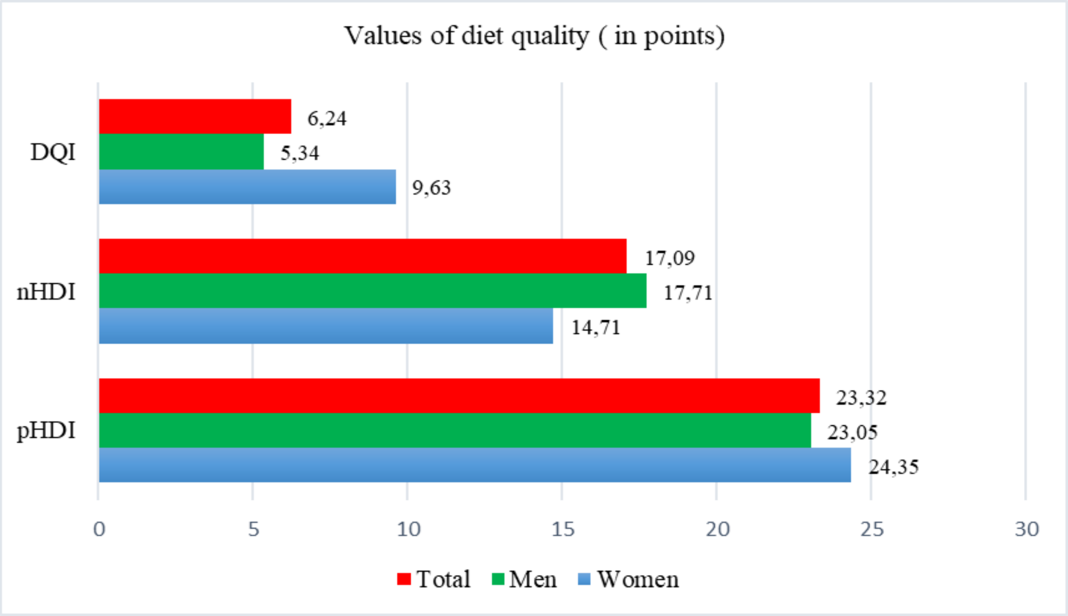

Figure 1. Comprehensive diet quality assessment
